# Supplementary material for: The Viral Protein Poly(A) Polymerase Catalytic Subunit Interacts with Guanylate-Binding Proteins 2 to Antagonize the Antiviral Ability of Targeting Ectromelia Virus
Source: Int J Mol Sci. 2023 Oct 30;24(21):15750. doi: 10.3390/ijms242115750 (PMC10648259; doi:10.3390/ijms242115750)
Supplement: Supplementary file 1 [file ijms-24-15750-s001.zip › ijms-2644244-supplementary.pdf]

SUPPLEMENTAL MATERIAL

Table S1. Oligo DNA and primers for construction and verification of ECTV lacking PAPL gene

Primer	Sequence (5'–3')	Note
PAPL_RNA13-F	CACCGTAGGAAGAGTATTCTGATC	guide sequences for PAMs
PAPL_RNA13-R	AAACGATCAGAATACTCTTCCTAC	
PAPL_13rv-Left	TGATATTCGTTCACACTGGGT	for PCR product validation
PAPL_13rv-Right	TCTGAACCAGTATCAGGAGTTTGT	
PAPL-RNA600-F	CACCGTTAATCCAAATATACGGTA	guide sequences for PAMs
PAPL_RNA600-R	AAACTACCGTATATTTGGATTAAC	
PAPL_600fw-Left	CGTTCATGGTATCCTGGCGA	for PCR product validation
PAPL_600fw-Right	CGTCGCAGTAATAGACAAGGC	
PAPL_RNA77-F	CACCGATATGATATTCGTTCACAC	guide sequences for PAMs
PAPL_RNA77-R	AAACGTGTGAACGAATATCATATC	
PAPL_77rv-Left	TCCGTGTATGTGGTCACTAATACA	for PCR product validation
PAPL_77rv-Right	GGAATCCTGATCAGAATACTCTTCC	
VSP Primer	CATATGCTTACCGTAACTTGAAAG	verify vector
PAPL-cris-P1	ACTCCGTTTTATATCAATACC	verification of PAPL gene sequences
PAPL-cris-P2	CCGATCGATATCAAGAACTG	

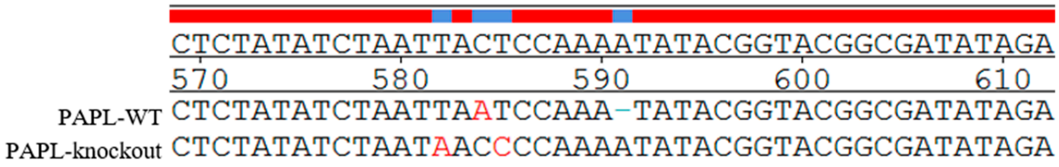

Figure S1. Results of the partial sequence alignment of PAPL-WT and PAPL-Knockout

Table S2. Primer sequences for plasmids construction in this study

Primer	Sequence (5'–3')	Note
HA-VITF3L-F	cggggtaccATGGATAATCTATTTACCTTTC	Kpn I
HA-VITF3L-R	ccgctcgagccTCATTTTAGAAGCAATTCTTTTAG	Xho I
HA-glutaredoxin-1-F	cggggtaccATGGCCGAGGAATTTGTAC	Kpn I
HA-glutaredoxin-1-R	ccgctcgagccTCAACAAGTTCTCAATACACC	Xho
HA-profilin-F	cggggtaccATGGCGGCCGAATGGCAT	Kpn I
HA-profilin-R	ccgctcgagccTTAATTACCAGTTGCTCGCACATTAG	Xho
HA-SEMA-F	cggggtaccATCGAATGGCATAAGTTTGAAACG	Kpn I
HA-SEMA-R	ccgctcgagggTTACATTTTAAGTATTTTTTTCATTCG	Xho
HA-KBTB1-F	cggggtaccATGAATAACAGCAGTGAATTAATTG	Kpn I
HA-KBTB1-R	ccgctcgagggTCAACTACCTATAAAACTCTCC	Xho
HA-PAPL-F	cggggtaccATGAATAGGAATCCTGATC	Kpn I
HA-PAPL-R	ccgctcgagccCTAAAATACAATTATATCTTTTTCG	Xho
HA-PAPL-D202G-F	ATATACGGTACGGCGGTATAGATATTCTTCAGAC	Base605
HA-PAPL-D202G-R	GTCTGAAGAATATCTATCTCGCCGTACCGTATAT	A-G
HA-PAPL-D204G-F	GGTACGGCGATATAGGTATTCTTCAGACTAATT	base611
HA-PAPL-D204G-R	AATTAGTCTGAAGAATCTCTATATCGCCGTACC	A-G
